# Informed consent in medically assisted procreation in Italy: medico-legal and ethical challenges shaping physician responsibility

**DOI:** 10.3389/frph.2026.1850431

**Published:** 2026-07-02

**Authors:** Costanza Raimondi, Pia Ferrari, Pietro Refolo, Antonio Oliva, Antonio G. Spagnolo

**Affiliations:** 1 Section of Bioethics and Medical Humanities, Department of Health Care Surveillance and Bioethics, Università Cattolica del Sacro Cuore, Rome, Italy; 2 Section of Legal Medicine, Department of Health Care Surveillance and Bioethics, Università Cattolica del Sacro Cuore, Rome, Italy; 3 Research Centre for Clinical Bioethics & Medical Humanities, Università Cattolica del Sacro Cuore, Rome, Italy; 4 Fondazione Policlinico Universitario A. Gemelli IRCCS, Rome, Italy

**Keywords:** artificial reproductive technology, counselling, informed consent, medically assisted procreation (MAP), physician responsibility

## Abstract

**Background:**

Informed consent represents a fundamental requirement in healthcare and is a key expression of patient autonomy. In the Italian context, its application in the field of medically assisted procreation (MAP) raises specific medico-legal and ethical questions. Recent developments, including updated MAP guidelines and evolving case law, further underscore the need to reflect on the specificities of informed consent in this setting.

**Objectives:**

This study aims 1) to trace the differences between informed consent in MAP and standard healthcare consent in Italy, and 2) to examine the medico-legal and ethical implications of these differences for physicians, whose obligations have come to extend beyond clinical information to encompass ethical, legal, psychological, and relational dimensions, particularly with regard to duties of information and professional liability.

**Materials and methods:**

This study adopts a qualitative, interdisciplinary approach based on a critical review of Italian legislation, case law, and bioethical and medico-legal literature. Sources were selected for relevance to MAP, with particular attention to complex scenarios such as embryo management after partner separation, post-mortem embryo transfer, and cryopreservation.

**Results:**

In Italy, informed consent in MAP differs substantially from standard healthcare consent because of its joint nature, its function beyond the therapeutic sphere, its temporal extension, and its partial irrevocability after fertilization. These features transform consent into a complex, processual act with long-term legal, ethical, and relational implications. As a result, the physician's role is not limited to the provision of technical information, but requires enhanced professional responsibility in ensuring adequate, comprehensible, and context-sensitive communication.

**Discussion:**

In this perspective, structured counselling, repeated informational processes, and careful documentation become essential not only to support autonomous decision-making, but also to prevent or mitigate medico-legal risks. These considerations highlight the need for specific professional training in communication and counselling within MAP settings, to adequately address the complexity of consent in this field.

## 1 Introduction

Informed consent is today recognized as a fundamental expression of personal autonomy in the healthcare context. In Italy, its legal foundation is commonly traced to the combined reading of Articles 2, 13 and 32 of the Italian Constitution, which respectively protect the inviolable rights of the person, personal liberty, and the right to health (Art. 2: The Republic recognises and guarantees the inviolable rights of the person, both as an individual and in the social groups where human personality is expressed. The Republic expects that the fundamental duties of political, economic and social solidarity be fulfilled; Art. 13: Personal liberty is inviolable (…); Art 32: The Republic safeguards health as a fundamental right of the individual and as a collective interest, and guarantees free medical care to the indigent. No one may be obliged to undergo any health treatment except under the provisions of the law. The law may not under any circumstances violate the limits imposed by respect for the human person) (1).

These principles have also been reinforced by international and European legal documents that emphasize the centrality of personal autonomy in medical decision-making, including the Convention on Human Rights and Biomedicine (Art 5: An intervention in the health field may only be carried out after the person concerned has given free and informed consent to it. This person shall beforehand be given appropriate information as to the purpose and nature of the intervention as well as on its consequences and risks. The person concerned may freely withdraw consent at any time) (2) and the Charter of Fundamental Rights of the European Union (Art 3 (2) (a): In the fields of medicine and biology, the following must be respected in particular: the free and informed consent of the person concerned, according to the procedures laid down by law) (3), both of which affirm the requirement of free and informed consent in the fields of medicine and biology. Through constitutional interpretation, judicial developments, and the growing awareness of personal autonomy, informed consent has progressively come to be understood as the necessary condition for any medical intervention, since any interference with the human body directly implicates personal freedom and dignity. This evolution reflects both medico-legal developments and bioethical perspectives, which converge in identifying informed consent as an expression of personal autonomy grounded in a communicative and relational model of care rather than in a purely paternalistic approach (4).

Informed consent, indeed, is not merely a formal authorization for medical intervention but the result of a process combining two essential elements: information and decision (5). As highlighted in medico-legal and bioethical scholarship, information and consent may be understood as two inseparable components of the same process, comparable to “two sides of the same coin”, since the validity of consent ultimately depends on the quality, adequacy, and comprehensibility of the information provided (4–6). The informational process therefore constitutes the necessary condition for the effective exercise of personal autonomy in medical decisions.

Prior to the introduction of a general statutory framework on informed consent in 2017, consent to medical interventions in Italy was governed through a plurality of sector-specific provisions. Such provisions governed, namely: mandatory and recommended vaccinations (Law 891/1939; Law 292/1963; Law 51/1966; Law 165/1991), compulsory health treatments (Law n.180/1978), voluntary termination of pregnancy (Law 194/1978), experimentation (Law 94/1998), removal and transplantations of cells, tissues and organs (Law 91/1999), and medically assisted procreation (Law 40/2004) (7–15).

The legal framework, established by Law No. 219/2017, guarantees that consent is personal, free, explicit, current, complete, and informed—characteristics that were already reflected, albeit in a fragmented manner, in the sector-specific provisions previously mentioned (16, 17).

Law No. 219/2017 provides, for the first time in the Italian legal system, a comprehensive and unified regulation of informed consent, establishing that no medical treatment may be initiated or continued without the free and informed consent of the person concerned (art. 1) (16). The same provision explicitly recognizes the patient's right to receive complete, updated, and comprehensible information regarding diagnosis, prognosis, risks, benefits, alternatives, and the consequences of refusal, thereby framing informed consent as a process grounded in communication and understanding. Within this framework, informed consent emerges not only as a legal prerequisite for medical intervention, but also as the cornerstone of the therapeutic relationship, where patient autonomy and physician responsibility intersect. Art. 1 of Law 219/2017 also states that information should be provided in a way that is understandable to the patient, and stresses that the time devoted to communication constitutes, in all respects, *time of care* (art. 1.8) (16). However, in the context of MAP in Italy, informed consent assumes distinctive characteristics that differentiate it from all other forms of medical consent and give rise to specific medico-legal and ethical challenges.

## 2 Objectives

The present study aims, first, to identify the distinctive features of informed consent in MAP compared with the standard healthcare consent in Italy, and second, to examine the medico-legal and ethical implications of these differences for physicians. Particular attention is devoted to the expansion of professional obligations beyond the mere provision of clinical information, encompassing ethical, legal, psychological, and relational dimensions, especially with regard to duties of information, counselling, and professional liability.

## 3 Materials and methods

This study adopted a qualitative and interdisciplinary research design combining doctrinal legal analysis with a review of the relevant bioethical and medico-legal literature. The aim of such methodological approach was to reconstruct and critically analyze the framework governing informed consent in MAP in Italy and to examine its implications for physician responsibility.

Primary sources included Italian statutory provisions, constitutional principles, judicial decisions and national guidelines relevant to informed consent and MAP. These materials were identified through legal database searches and selected according to their relevance to the study objectives, with particular attention to issues concerning withdrawal of consent, embryo transfer following partner separation or death, cryopreservation, and physician responsibility. The analysis focused exclusively on the Italian legal framework. References to international and supranational sources were considered solely when necessary to contextualize the topic.

Secondary sources were identified through searches conducted in PubMed and Google Scholar between December 2025 and February 2026. Search terms included combinations of “informed consent”, “medically assisted procreation”, “assisted reproductive technology”, “physician liability”, “bioethics”, and “Italy”. Peer-reviewed publications addressing informed consent in MAP within the Italian legal framework were included, while publications unrelated to informed consent in MAP or focused exclusively on technical reproductive outcomes were excluded.

The collected legal and scholarly sources were analyzed using a qualitative and interpretative approach. The analysis focused on two main thematic axes: (1) the distinctive medico-legal characteristics of informed consent in MAP compared with standard healthcare consent; and (2) the implications of these characteristics for physicians’ duty of information, particularly in relation to professional responsibility and liability.

This methodological approach enabled an integrated assessment of legal norms, judicial interpretations, and bioethical perspectives, providing a comprehensive understanding of the specific features and implications of informed consent in MAP.

## 4 Results

### 4.1 Medico-legal characteristics and ethical concerns regarding MAP consent

Within this landscape, the discipline of MAP, regulated by Law No. 40/2004 (15), introduced a markedly different kind of informed consent—with distinctive medico-legal and ethical implications—which differed greatly from the other provisions at the time and differs still from the legal framework provided by Law 219/2017. The peculiar nature of consent in MAP in Italy can be better understood through four interrelated characteristics that distinguish it from traditional therapeutic consent: 1) its joint character, 2) its function beyond the strictly therapeutic sphere, 3) its temporal extension across multiple procedural phases, and 4) its partial irrevocability after fertilization.

First, consent is not expressed by an individual patient alone, but by the couple jointly accessing the reproductive pathway, regardless of which partner is directly affected by infertility (Art. 6.3: The will of both individuals to access medically assisted procreation techniques is expressed in writing, jointly, in the presence of the physician who is responsible for the facility) (15). This necessarily follows from Article 5 of Law 40/2004, which allows access to MAP only to couples—namely, “adult couples of different sexes, married or cohabiting, of potentially fertile age, both living”—thereby excluding single individuals (15). The joint character of consent in MAP highlights a fundamental departure from the general model of informed consent in healthcare: while ordinary medical consent is expressed by an individual patient and primarily protects that individual's bodily integrity and self-determination, MAP consent is necessarily formulated by the couple as a unit. In this sense, consent is not only solely individual but intrinsically relational, as it reflects a shared decision-making process concerning a common reproductive project. This joint dimension may introduce asymmetries, dependencies, and future conflicts between partners, particularly when their intentions diverge over time. Consent therefore acquires a partly constitutive function, insofar as it contributes to the legal recognition of a shared parental intent and to the structuring of a reproductive project whose implications extend beyond the individual sphere (17, 18).

A closely related aspect concerns the fact that MAP consent extends beyond the therapeutic sphere. Unlike standard medical interventions, which are primarily aimed at diagnosing, treating, or preventing disease in a patient, MAP involves the intentional creation of a new life and the establishment of parental relationships. Therefore, consent functions not only as authorization for a medical procedure, but also as the foundation of a reproductive project that generates legal and ethical consequences in terms of filiation, parental responsibility, and family relationships. In this perspective, the role of consent shifts from regulating an intervention on the patient's body to governing decisions that produce prospective and relational effects. This raises the broader question of whether the traditional model of informed consent is fully adequate to address decisions involving the intentional creation of a new life, the establishment of parental roles, and the assumption of long-term responsibilities extending beyond the clinical context.

Another distinctive feature concerns the temporal dimension of consent. In the Italian healthcare context, informed consent typically accompanies interventions whose effects are temporally limited. MAP, by contrast, unfolds through multiple phases that may be separated in time (i.e., ovarian stimulation, gamete retrieval, fertilization, cryopreservation, and embryo transfer); in particular, since embryo cryopreservation has been allowed, the temporal dimension of consent has been significantly expanded (19). In the Italian legal framework, cryopreserved embryos that are not subsequently transferred into the woman's uterus cannot be destroyed, donated to other couples or used for research. As a result, cryopreservation may lead to situations in which embryos remain preserved for an indefinite period. As highlighted in case-law analyses, this temporal fragmentation means that consent expressed at one stage may generate consequences long after the original relational context has changed (20). Situations involving partner separation have demonstrated how earlier consent may conflict with later dissent, creating complex legal and ethical dilemmas, as discussed in the next paragraphs.

A final distinctive element is the irrevocability of consent following fertilization, which represents a clear deviation from the principle of revocability that is a cornerstone of Italian healthcare consent. Pursuant to Article 6, paragraph 3, consent may be withdrawn by either member of the couple only until the fertilization of the oocyte (Art 6.3: Consent may be withdrawn by either of the individuals until the moment of fertilization of the oocyte) (15). This provision creates a significant medico-legal and ethical tension with the principle of revocability traditionally associated with informed consent, raising questions regarding the limits of autonomy when decisions produce irreversible consequences over time. Scholarship has observed that this feature gives Italian MAP consent a distinctive legal nature, placing it between traditional healthcare consent and a legally relevant procreative act (17). The medico-legal relevance of this rule becomes particularly evident in cases of partner separation, where the original consent may continue to produce legal effects despite changes in the couple's relationship (18, 21).

Indeed, both Court Rulings and the MAP Guidelines issued by the Italian Minister of Health have established that a woman can proceed with uterine transfer of the already fertilized embryo even when the couple has separated or the male partner has died, without any temporal limitation, potentially resulting in children being born years after their father's death (22). The possibility that a child may be born after the father's death, as well as the possibility that a child be born to parents who have already separated and where the father no longer wishes to pursue the reproductive project, reveal the far-reaching consequences of the irrevocability of consent in MAP (23).

These scenarios raise fundamental ethical questions concerning autonomy, responsibility, and the legitimacy of decisions that continue to produce effects in radically altered relational contexts (21). From this perspective, consent in MAP acquires a prospective and relational dimension, extending its implications beyond the immediate subjects involved and shaping responsibilities toward future persons. Decisions regarding fertilization, embryo preservation, and embryo transfer may directly influence the circumstances under which a child is born, including situations in which one parent is absent due to separation or death. While the legal framework primarily focuses on the validity of consent between the partners, the ethical implications extend to the interests and welfare of the future child. This raises the question of whether consent expressed within a previous relational context can still be considered an adequate expression of a shared parental intent when the circumstances in which it was originally given have substantially changed.Physician role and responsibility in collecting MAP consent.

Given these distinctive features of the legal framework governing MAP in Italy, the physician's role far extends beyond the provision of technical information about procedures and risks. Physicians are required to fulfil broader informational and communicative duties, particularly with regard to the ethical and legal dimensions of MAP procedures and the validity of informed consent.

Article 6 of Law No. 40/2004 expressly provides that couples must receive comprehensive information not only about the medical aspects of the procedure but also about its bioethical implications and legal consequences (Art 6.1: The physician provides the individuals referred to in Article 5 with detailed information on the methods, the bioethical issues, and the possible health and psychological side effects resulting from the application of such techniques, as well as on the likelihood of success and the risks involved, and on the related legal consequences for the woman, the man, and the unborn child) (15). The law therefore requires that this information be provided before consent is obtained and in a way that enables a conscious and informed reproductive decision. This is true for all informed consent processes: both medico-legal and bioethical scholarship stress that the adequacy of consent depends not only on the quantity of information provided, but on the quality of communication, which must be tailored, comprehensible, and grounded in a relationship of trust between physician and patient, as also affirmed by the Italian National Bioethics Committee (4). However, in the context of MAP, this requirement acquires particular relevance due to the complexity and long-term consequences of the decisions involved.

To these already expanded responsibilities must be added the peculiar vulnerability often experienced by couples undergoing MAP. Fertility treatments are frequently associated with strong expectations and emotional distress, factors that may significantly influence decision-making processes. For this reason, the adequacy of consent cannot be evaluated solely in terms of information provided but must also take into account the patient's actual understanding (4, 17). This further increases in the physician's responsibility, requiring not only the provision of accurate information (medical, as well as legal and ethical), but also advanced communicative and relational skills capable of supporting vulnerable patients throughout the decision-making process.

In this context, it is important to distinguish between different dimensions of professional obligation. Information refers to the duty to provide complete, updated, and comprehensible clinical and legal information relevant to MAP procedures, consistent with Article 1 of Law No. 219/2017 and Article 6 of Law No. 40/2004. Communication concerns the manner in which such information is conveyed and understood within the physician-patient relationship. Counselling, by contrast, should be understood as a structured supportive process aimed at facilitating informed reproductive decision-making in ethically and emotionally complex situations. Finally, professional liability concerns the medico-legal consequences that may arise when deficiencies in these duties compromise the validity or effectiveness of informed consent.

Moreover, consent in MAP is inherently relational and jointly expressed, a feature that may generate asymmetries and potential conflicts between partners. Since consent to MAP may also produce legal effects that persist over time (17, 18), the physician's duty to inform extends beyond the explanation of medical procedures and risks to include discussion of foreseeable—though undesirable—future implications, such as separation, disputes concerning embryos, and the consequences of the irrevocability of consent following fertilization (20). However, this does not imply that physicians are expected to predict or manage all future interpersonal conflicts between partners. Rather, their responsibility lies in ensuring that patients receive adequate and comprehensible information regarding the consequences directly connected to MAP procedures. In this sense, the informational process may also perform a preventive function with respect to potential disputes, and failure to adequately address these aspects may expose physicians to allegations of defective consent, even in the absence of technical malpractice.

This aspect is particularly relevant from a medico-legal perspective, as litigation in the field of MAP frequently concerns not the technical execution of the procedure, but rather the validity and adequacy of the consent process. Disputes often arise when one partner claims insufficient awareness of the consequences connected to fertilization and embryo use, especially after relational changes such as separation (20). Accordingly, medico-legal evaluation focuses on the quality, comprehensibility, and completeness of the information provided, rather than on the technical execution of the procedure. In this regard, adequate documentation and counselling may constitute crucial components of good practice.

Documentation plays a particularly important evidentiary role: since disputes may arise long after fertilization, accurate records of counselling, information provided, and the couple's expressed understanding become central to the reconstruction of the consent process. Medico-legal scholarship has observed that, in MAP, consent is more appropriately understood as a process rather than a single formal act (24, 25).

Another relevant dimension concerns causation, namely whether inadequate counselling may have affected the patient's ability to make a fully informed decision. In MAP disputes, alleged harm is often relational or existential rather than physical, such as unwanted parenthood—for the father—or a perceived loss of reproductive autonomy. In such cases, medico-legal assessment may revolve around whether clearer or more comprehensive counselling could reasonably have influenced the decision-making process (17). In this sense, counselling may be viewed not only as an ethical requirement but also as a medico-legal safeguard, since inadequate counselling may expose physicians to allegations of defective consent even in the absence of technical malpractice.

These aspects suggest that physician competence in MAP in Italy cannot be limited to clinical expertise, but should include training in communication, ethical and legal competences, and structured counselling practices, in order to ensure both the validity of consent and the prevention of future disputes.

For these reasons, physician responsibility in MAP can be understood as requiring enhanced professional diligence including structured counselling, repeated information process, and tailored communication strategies. While clinicians are not expected to provide ethical or legal advice, they must ensure that patients are aware of the relevant ethical implications and of the legal framework governing MAP, together with its practical implications.

Overall, structured counselling and careful documentation should be regarded as key elements of good professional practice and risk prevention in MAP settings.

## 5 Discussion

Taken together, these elements show that consent in MAP in Italy cannot be regarded merely as an authorization for medical procedure. Its joint nature, its projection beyond the therapeutic sphere, its temporal extension, and its partial irrevocability after fertilization transform consent into a complex and processual act situated at the intersection of autonomy, relationality, and long-term responsibility.

The practical implications of this transformation have become increasingly evident in light of recent case law and, in particular, the updated MAP Guidelines issued by the Italian Ministry of Health. These developments allow the use of cryopreserved embryos even after the dissolution of the couple or the death of the male partner, thereby producing scenarios in which the effects of consent persist independently of the evolving will and relational context of the individuals involved. Such situations—post-mortem reproduction and post-separation embryo transfer—highlight the limits of traditional consent models and generate ethically and legally problematic outcomes that directly affect clinical practice.

In this context, the physician's role inevitably assumes a central and expanded function. Pursuant to the applicable legal framework, physicians are required not only to provide medical information, but also to ensure that patients are adequately informed about the legal and ethical implications of their decisions, including foreseeable future scenarios that may arise from the application of MAP techniques. The consent process must therefore be understood as a structured and ongoing communicative activity, rather than a single formal act.

From a practical perspective, this entails the need to strengthen several core components of professional practice. First, documentation must be extremely accurate, comprehensive, and capable of reflecting the entire decision-making process, given its crucial evidentiary role in potential litigation. From a medico-legal standpoint, this means that professional diligence in MAP cannot be assessed exclusively based on technical correctness of the procedure. Increasingly, liability assessment may also depend on the physician's ability to demonstrate that the informational process was comprehensive, comprehensible, appropriately documented, and consistent with the communicative standards established by Law No. 219/2017. Second, specific training in ethical reasoning and legal awareness is required, in order to adequately address the hybrid nature of MAP consent and its long-term implications. Third, physicians should be trained to master enhanced communication skills, ensuring that information is not only provided but effectively understood, particularly in situations characterized by emotional vulnerability and complex decision-making. Counselling should be regarded as an integral component of the consent process. Structured counselling allows physicians to address relational dynamics, asymmetries between partners, and foreseeable future conflicts, thereby supporting genuinely informed and responsible decision-making. While the present contribution adopts a theoretical perspective, future studies could complement this analysis by investigating the topic in real clinical settings.

In light of these considerations, strengthening physician training in communication and counselling skills, medico-legal knowledge, ethical awareness, and accurate documentation practices emerge as necessary steps to ensure both the validity of consent and the reduction of potential disputes, thereby enabling physicians to fully meet the responsibilities and duties that are asked of them by the current legal framework.
